# Genetic and molecular correlates of cortical thickness alterations in adults with obsessive–compulsive disorder: a transcription–neuroimaging association analysis

**DOI:** 10.1017/S0033291724001909

**Published:** 2024-09

**Authors:** Da Zhang, Changjun Teng, Yinhao Xu, Lei Tian, Ping Cao, Xiao Wang, Zonghong Li, Chengbin Guan, Xiao Hu

**Affiliations:** 1 Department of Radiology, The Affiliated Brain Hospital of Nanjing Medical University, Nanjing, Jiangsu, China; 2 Department of Medical Psychology, The Affiliated Brain Hospital of Nanjing Medical University, Nanjing, Jiangsu, China

**Keywords:** Allen Human Brain Atlas, cortical thickness, gene expression, obsessive–compulsive disorder, transcriptome

## Abstract

**Background:**

Although numerous neuroimaging studies have depicted neural alterations in individuals with obsessive–compulsive disorder (OCD), a psychiatric disorder characterized by intrusive cognitions and repetitive behaviors, the molecular mechanisms connecting brain structural changes and gene expression remain poorly understood.

**Methods:**

This study combined the Allen Human Brain Atlas dataset with neuroimaging data from the Meta-Analysis (ENIGMA) consortium and independent cohorts. Later, partial least squares regression and enrichment analysis were performed to probe the correlation between transcription and cortical thickness variation among adults with OCD.

**Results:**

The cortical map of case-control differences in cortical thickness was spatially correlated with cortical expression of a weighted combination of genes enriched for neurobiologically relevant ontology terms preferentially expressed across different cell types and cortical layers. These genes were specifically expressed in brain tissue, spanning all cortical developmental stages. Protein–protein interaction analysis revealed that these genes coded a network of proteins encompassing various highly interactive hubs.

**Conclusions:**

The study findings bridge the gap between neural structure and transcriptome data in OCD, fostering an integrative understanding of the potential biological mechanisms.

## Introduction

Obsessive–compulsive disorder (OCD) is a heterogeneous psychiatric condition characterized by persistent intrusive thoughts (obsessions) and compulsive behaviors (Schulze, Kathmann, & Reuter, 2018). OCD is estimated to affect 1–3% of the adult population over their lifetime and is related to significant personal impairment and socioeconomic burden (Stein et al., 2019). Due to the unsatisfactory response rates of first-line treatments against OCD and its elusive etiology, there is an urgent need for further research into the biological underpinnings of the disorder to identify biomarkers and support prediction, leading to effective treatments (Goodman, Storch, & Sheth, 2021; Robbins, Vaghi, & Banca, 2019).

The brain, the material foundation for cognition and behavior, undergoes rapid development via a dynamic process with region-specific patterns (Bethlehem et al., 2022; Qiu, Zhang, Kennedy, & Lee, 2021). Neuroimaging studies have revealed that cortical thickness is a valuable measure to assess neuroanatomical patterns associated with neurodegeneration, conferring particular sensitivity for detecting subtle, previously unrecognized anatomical changes (Amlien et al., 2016; Sydnor et al., 2021; Walhovd, Fjell, Giedd, Dale, & Brown, 2017). Previous neuroimaging studies on OCD have reported altered cortical thickness, primarily involving the fronto-limbic and fronto-parietal regions (Kühn et al., 2013; Shin et al., 2007), associated with cognitive control and emotional regulation. A recent meta-analysis on OCD patients identified altered cortical thickness in the superior and inferior frontal regions, precentral, posterior cingulate, middle temporal, inferior parietal, and precuneus gyri (Fouche et al., 2017). These findings indicate the role of cortical abnormalities in OCD pathophysiology and their association with clinical symptoms. However, the molecular mechanisms underlying such brain structural alterations remain unknown.

Genetic factors play a significant role in brain development, with various disease-related imaging-derived phenotypes demonstrating high heritability (Arnatkeviciute, Fulcher, Bellgrove, & Fornito, 2021; Fornito, Arnatkevičiūtė, & Fulcher, 2019; Pizzagalli et al., 2020; Thompson et al., 2001). Similarly, OCD is a heritable disorder involving multiple genetic alterations and possibly affecting different molecular pathways, resulting in heterogeneous phenotypes (Alemany-Navarro et al., 2020; Mahjani, Bey, Boberg, & Burton, 2021; Monzani, Rijsdijk, Harris, & Mataix-Cols, 2014). Thus, genetic factors can contribute to alterations in molecular pathways that result in the structural abnormalities seen in OCD. While traditional imaging genetics analyses have underscored the substantial influence of genetic factors on a fewer of brain phenotypes associated with OCD, the intermediary mechanisms involved remain undefined (Atmaca et al., 2011; Gassó et al., 2015; Sinopoli et al., 2020; Wu et al., 2013). Gene expression constitutes the most essential molecular mechanism by which genes govern the differentiation, development, and functionality of brain cells and tissues (Naumova, Lee, Rychkov, Vlasova, & Grigorenko, 2013).

Recent developments in neuroimaging and transcriptomics make it possible to directly investigate the association of neuroimaging-detected alterations with gene expression patterns across distinct brain regions (Atmaca et al., 2011; Grünblatt, Hauser, & Walitza, 2014). The whole-brain atlas of gene expression derived from the Allen Human Brain Atlas (AHBA) database has proven instrumental for delving into disease-related gene expression and the molecular basis of brain abnormalities in various psychiatric disorders (M. Hawrylycz et al., 2015; Li et al., 2021; Morgan et al., 2019; Sun et al., 2023; Sunkin et al., 2013; Xue et al., 2023), offering new avenues for deciphering the molecular basis of OCD. Saraiva, Sato, and Cappi (2022) illustrate this approach, proposing a model associating connectome alterations in OCD with AHBA expression data to develop personalized therapeutic mechanisms.

**Figure 1.**
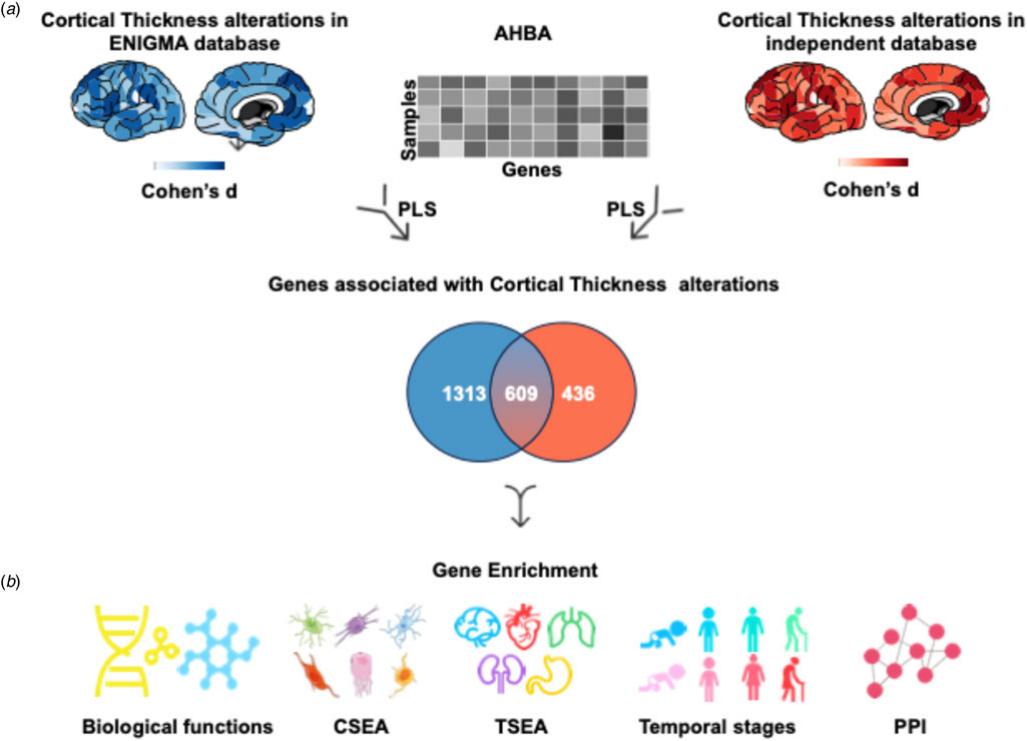
Schematic workflow of the analytical framework. (*a*) PLS regression was conducted to link cortical thickness abnormalities of OCD with gene expression data, the overlapped genes were identified by taking the intersection of the significant gene list from the first PLS component (PLS1) from both ENIGMA and independent database. (*b*) Enrichment analyses were performed subsequently on the overlapped genes. AHBA, Allen Human Brain Atlas; CSEA, cell-type-specific expression analysis; OCD, obsessive–compulsive disorder; PPI, protein–protein interaction; PLS, partial least squares; TSEA, tissue-type-specific expression analysis.

Based on this innovative landscape, the present study attempts to bridge the gap between cortical thickness alterations within OCD and underlying gene expression patterns, employing a novel transcriptome–neuroimaging spatial correlation analysis to pinpoint genes whose expression levels correlate with cortical thickness changes in OCD. Through comprehensive enrichment analyses, these genes could be mapped to molecular pathways, cellular classifications, cortical layers, and protein–protein interactions (PPIs), providing a multi-faceted view of the pathophysiology of OCD. Figure 1 illustrates our analytical approach, underscoring the potential of integrating neuroimaging and genomic data to elucidate the complex biological mechanisms of psychiatric disorders. This could pave the way for more effective and personalized treatment modalities.

### ENIGMA participants

For the transcriptional analyses, we used publicly accessible multi-site summary statistics published by the ENIGMA Consortium (available through the ENIGMA Toolbox: https://github.com/MICA-MNI/ENIGMA) (Larivière et al., 2021). The ENIGMA-OCD working group comprises 38 datasets from 27 international research institutions, including neuroimaging and clinical data from OCD patients and the healthy controls (HCs) (Boedhoe et al., 2018). Our analysis was limited to adults to account for age-related variation in cortical structure. We analyzed data from 2934 individuals, including 1498 OCD patients and 1436 HCs. Our analyses were based on covariate-adjusted case-control differences, employing random-effects meta-analyses of *z*-scored effect sizes (Cohen's *d*) for cortical thickness. It should be noted that the Desikan–Killiany Atlas (Desikan et al., 2006) (consisting of 68 cortical regions) is the sole representation of the ENIGMA datasets. However, we converted the data to the Human Connectome Project (HCP) multi-modal parcellation 1.0 atlas (180 parcellations per hemisphere) for the subsequent analyses (Glasser et al., 2016). This atlas enhances our analysis by providing a higher resolution of brain structure, enabling more precise localization of cortical changes associated with OCD. Detailed information regarding magnetic resonance imaging (MRI) acquisition and sample demographics has been previously reported (Boedhoe et al., 2018).

### Independent dataset participants

Cortical thickness effect sizes (Cohen's *d*) were *z*-scored and validated within an independent cohort of 57 OCD patients and 57 well-matched HCs. OCD patients were enrolled consecutively from inpatient and outpatient units of the Affiliated Brain Hospital of Nanjing Medical University. HCs with no history of severe medical or neuropsychiatric illnesses were recruited from the local community. This study was approved by the ethics committee of the Affiliated Brain Hospital of Nanjing Medical University, and all the participants provided written informed consent. Demographic and clinical characteristics of the independent sample are presented in online Supplementary Table S1. Details of the inclusion and exclusion criteria, MRI data acquisition parameters, and cortical thickness measurement methods are provided in the online Supplementary materials.

### Assessment of regional gene expression

Brain-wide gene expression data were procured from the publicly accessible AHBA database (http://www.brain-map.org) (M. J. Hawrylycz et al., 2012; M. Hawrylycz et al., 2015), which provides comprehensive and high-resolution coverage of normalized microarray expression data across six donated post-mortem brains (online Supplementary Table S2). The original expression data, which encompasses more than 20 000 genes from 3702 spatially distinct brain tissue samples, were processed with the following analytic pipeline (Arnatkeviciute, Fulcher, & Fornito, 2019): (1) probe-to-gene annotations were confirmed using Re-annotator (Arloth, Bader, Röh, & Altmann, 2015); (2) probes falling below the background noise intensity in over half of the samples were filtered out; (3) the most RNA-seq-correlated probe for each gene was chosen; (4) samples were assigned to the HCP-360 atlas within a 2 mm Euclidean distance of the parcel (Glasser et al., 2016); (5) expression measures were normalized with a scaled robust sigmoid for each participant; and (6) gene set filtering was performed according to differential stability (DS). We recruited the top 50% of genes with the highest DS values to conserve gene expression across subjects for transcription–neuroimaging spatial correlations (M. Hawrylycz et al., 2015). To assess the impact of various DS threshold selections, we performed sensitivity analyses using two alternative DS cutoff thresholds: the upper 40% and 60%. The counts of residual probes and genes at each processing stage are presented in online Supplementary Fig. S1. Finally, the transcriptional level of each gene at each brain region was determined to obtain a matrix (360 regions × 5013 gene expression levels) for subsequent analyses.

### Regional cortical thickness alterations and gene expression

The correlation between the transcriptional activity of all 5013 genes and regional changes in cortical thickness (represented by Cohen's *d*-values from 360 cortical areas) was examined with partial least squares (PLS) regression (Abdi & Williams, 2013). The *z*-score normalized gene expression matrix (360 regions × 5013 genes) of this model predicts the *z*-score normalized Cohen's *d*-values of cortical thickness variation (response variable). The primary component of the PLS regression result (PLS1) represents a linear combination of gene expression values exhibiting the most significant correlation with regional alterations in cortical thickness. To validate the significance of PLS1 in elucidating the covariance between cortical thickness changes and genome-wide expression, we performed permutation testing using 5000 iterations with spherical rotations to control for spatial autocorrelation (Li et al., 2021; Romero-Garcia et al., 2020; Váša et al., 2018). Subsequently, the PLS weight of each gene was evaluated for variability using 5000 bootstrap replications. Then, a *z*-score was computed for each gene using the ratio of its PLS weight to the standard error from bootstrapping (online Supplementary File 1) (Morgan et al., 2019). Only genes with significant correlations (Benjamini–Hochberg false-discovery rate [BH-FDR] procedure *p* < 0.05) were considered for additional analysis (Bigdeli et al., 2016). This approach yielded two gene lists: genes depicting a significant positive correlation with regional cortical thickness changes (PLS1+) and another with genes showing a significant negative correlation (PLS1−). Transcriptome–neuroimaging association analysis was performed separately for Cohen's *d*-values map from ENIGMA and independent datasets.

### Enrichment analyses

Various enrichment analyses were performed for significant PLS+/− genes associated with OCD pathogenesis. First, functional annotation was performed using WebGestalt (https://www.webgestalt.org/) (Liao, Wang, Jaehnig, Shi, & Zhang, 2019). Gene ontology (GO) was used to ascertain biological functions, encompassing molecular functions (MFs), biological processes (BPs), and cellular components (CCs) (Thomas et al., 2022). The Kyoto Encyclopedia of Genes and Genomes (KEGG) helped identify relevant biological pathways (Kanehisa, Furumichi, Tanabe, Sato, & Morishima, 2017). Second, online tools for Tissue-type-Specific Expression Analysis (TSEA) and Cell-type-Specific Expression Analysis (CSEA) (http://genetics.wustl.edu/jdlab/tsea/ and http://genetics.wustl.edu/jdlab/csea-tool-3/) helped conduct specialized analyses for tissue and temporal expression (Dougherty, Schmidt, Nakajima, & Heintz, 2010; Xu, Wells, O'Brien, Nehorai, & Dougherty, 2014). Third, cell-type-specific expression was analyzed to determine the specific cell types associated with the gene set. Gene expression data for specific cell types in the human brain, including neurons, astrocytes, oligodendrocytes, microglia, and macrophages, were obtained from the CellMarker database (Zhang et al., 2019). Cortical layer enrichment was executed with marker genes from a previous transcriptomic study (He et al., 2017). Fourth, PPI analysis was performed using STRING v12.0 (https://string-db.org/) (Szklarczyk et al., 2023). Lastly, we examined the overlap between genes associated with alterations in cortical thickness in OCD patients identified in the present study and OCD-associated genes within the MalaCards (https://www.malacards.org/) and Obsessive–Compulsive Disorder databases (http://alpha.dmi.unict.it/ocdb/) (Privitera et al., 2015; Rappaport et al., 2017). Further details of these enrichment analyses are provided in the online Supplementary materials.

### Validation analysis

The primary analysis focused on genes in the top 50% of the highest differential stability scores to obtain consistent expression patterns across six donors. To ensure robustness in processing brain gene expression data, we also explored the effects of varying DS cutoff thresholds, the top 40% and 60% (Chen et al., 2022; Sun et al., 2023). Gene selection was based on the intersection of findings from two independent transcriptome–neuroimaging association analyses. This intersection approach strengthens the confidence in the identified genes, combining results from different analyses, thereby enhancing the reliability and robustness of the findings. Considering the potential confounding effects of spatial autocorrelation, a spin based method permutation tests (5000 times) were performed to preserve the spatial covariance structure of the data (https://github.com/frantisekvasa/rotate_parcellation) (Váša et al., 2018).

## Results

### Genes associated with cortical thickness alterations in OCD patients

**Figure 2.**
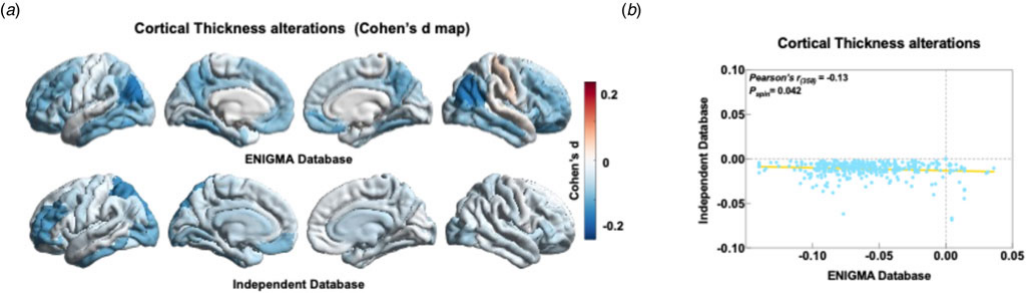
Cortical thickness alterations between patients with OCD and HCs. (*a*) Cohen's *d* maps indicating case-control differences in cortical thickness. (*b*) Region-wise Pearson's correlation between Cohen's *d* maps from the two datasets (Pearson's *r* = −0.13, *p*_spin_ = 0.042). HC, healthy control; OCD, obsessive–compulsive disorder.

**Figure 3.**
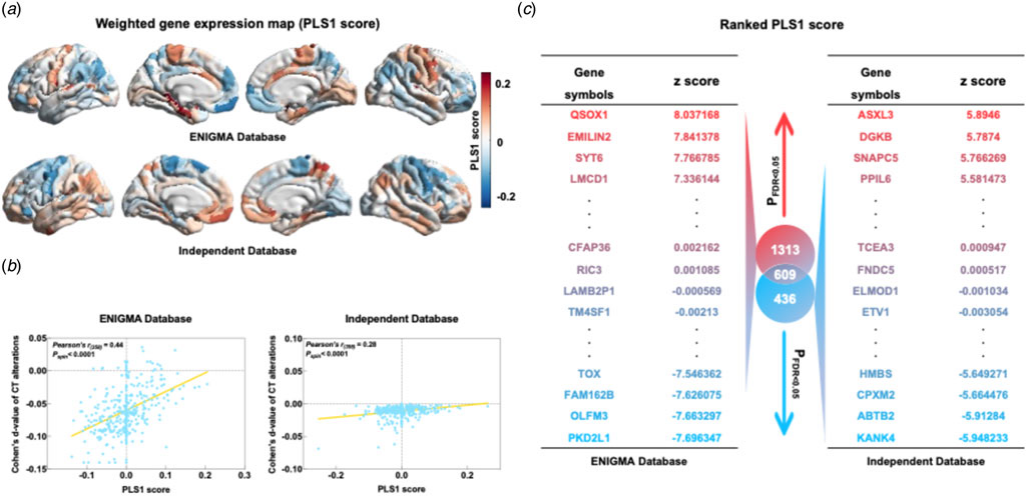
Gene expression profiles related to case-control differences in cortical thickness. (*a*) Cortical map of regional PLS1 scores. (*b*) Scatterplot of regional PLS1 scores (weighted sum of 5013 gene expression scores) *v*. case-control differences in regional cortical thickness. (*c*) Ranked PLS1 gene expression weights and the overlapped genes of the two datasets (609 genes are overlapped).

The study possessed sufficient statistical power to identify subtle cortical abnormalities in OCD. The Cohen's *d*-values map, produced via the ENIGMA Toolbox (Larivière et al., 2021), illustrates case-control differences in cortical thickness across databases (Fig. 2a). The spatial correlation analysis revealed a slight negative correlation (Pearson's *r*_(358)_ = −0.13, *p*_spin_ = 0.042, Fig. 2b) between the Cohen's *d*-value map of case-control cortical thickness differences within the two datasets. In the PLS regression analysis, gene expression in brain regions demonstrated significant correlations with inter-group differences in cortical thickness (ENIGMA dataset, Pearson's *r*_(358)_ = 0.44, *p*_spin_ < 0.0001; independent dataset, Pearson's *r*_(358)_ = 0.28, *p*_spin_ < 0.0001, Fig. 3a, b), utilizing a DS threshold of 50%. By ranking the normalized weights of the first PLS component using univariate *z*-tests (*p* < 0.05, FDR-corrected), we identified 1922 genes significantly connected with case-control cortical thickness changes in the ENIGMA dataset (PLS1 explained 28.3% of the variance) and 1045 in the independent dataset (PLS1 explained 22.5% of the variance), of which 609 genes overlapped (Fig. 3c, online Supplementary File 1). Significant intersections were also observed between the genes in the principal analysis and those identified using the two alternative DS thresholds of 40% (with an overlap ratio of 98.93%) and 60% (with an overlap ratio of 82.65%) (online Supplementary File 2).

### Enrichment for biological functions

**Figure 4.**
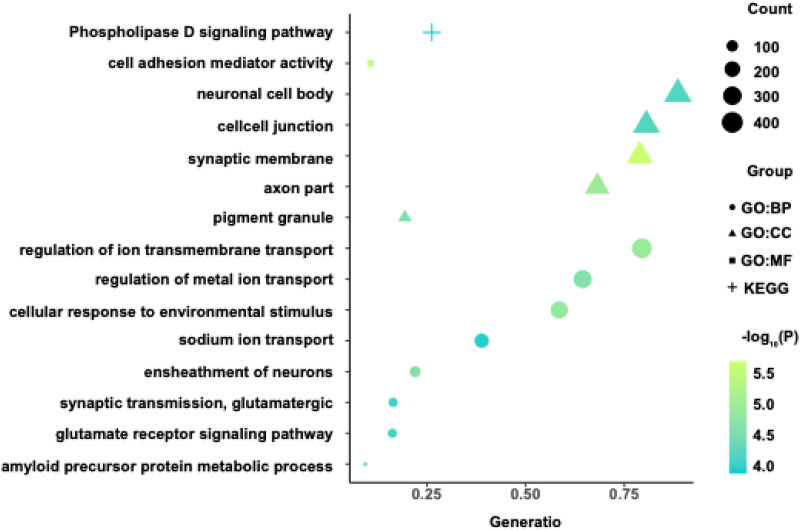
Biological function analysis the genes associated with cortical thickness alterations in OCD patients. Only items passed the BH-FDR-corrected threshold (*p* < 0.05) are displayed in the bubble chart. The *x*-axis shows the gene ratio of each item, the *y*-axis shows GO terms or KEGG pathway, the bubble shapes representing different categories, the bubble size indicates the intersecting number of genes in the category, and the color bar represents the −log_10_(*P*). BH-FDR, Benjamini–Hochberg false-discovery rate; BP, biological process; CC, cellular component; GO, gene ontology; KEGG, Kyoto Encyclopedia of genes and genomes; MF, molecular function; OCD, obsessive–compulsive disorder.

The GO and KEGG pathways were aligned with the overlapped gene list using WebGestalt (Liao et al., 2019). The most significant GO biological function and KEGG pathway enrichment terms are presented in Fig. 4. The genes associated with cortical thickness alterations were enriched for BPs, including regulation of ion transport across cell membranes, cellular responses to environmental stimuli, regulation of metal ion transport, neuron ensheathment, amyloid precursor protein metabolism, glutamate receptor signaling, glutamatergic synaptic transmission, and sodium ion transport. MFs enriched among these genes comprised of cell adhesion mediator activity, while the enriched CCs included synaptic membranes, axon segments, pigment granules, cell–cell junctions, and neuronal cell bodies. KEGG pathway analysis indicated significant cortical thickness alteration-related gene enrichment in the phospholipase D signaling pathway.

### Tissue-specific and temporal-specific expression analyses

**Figure 5.**
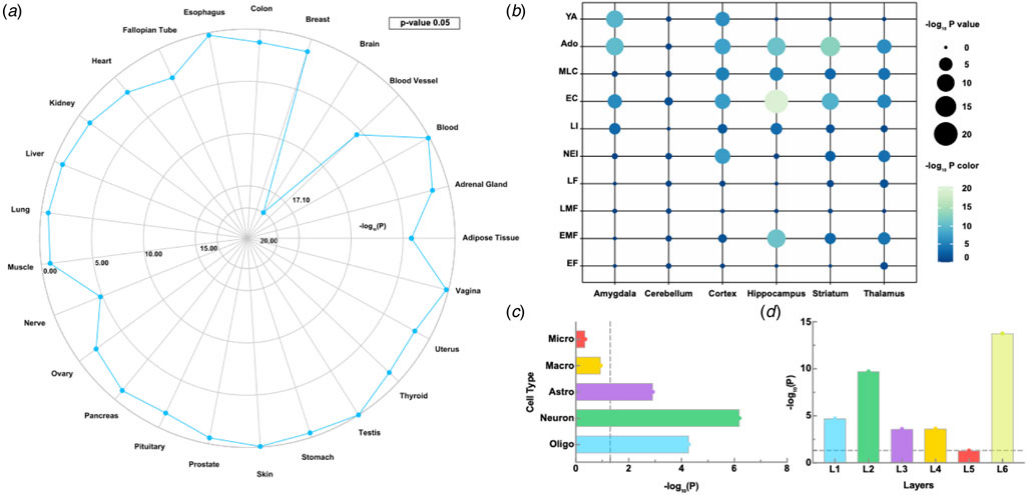
Specific expression analysis of the genes associated with cortical thickness alterations in OCD patients. (*a*) Tissue-type-specific expression analysis. (*b*) Cell-type-specific expression analysis. (*c*) Cortical layer enrichment analysis. (*d*) Developmental gene expression enrichment analysis. Ado, adolescence; EC, early childhood; EF, early fetal; EMF, early and mid-fetal; LF, late fetal; LI, late infancy; LMF, late and mid-fetal; MLC, middle and late childhood; NEI, neonatal and early infancy; OCD, obsessive–compulsive disorder; YA, young adulthood.

TSEA demonstrated that the 609 genes expressed specific brain tissue expression (*p* = 8.00 × 10^−14^, BH-FDR-corrected) under a pSI threshold of 0.05 (Fig. 5a and online Supplementary File 3). We conducted a temporal-specific expression analysis using the CSEA tool (Xu et al., 2014), comparing the selected gene list to developmental enrichment profiles. Developmental gene expression analysis indicated that these genes were expressed in the brain from early/mid-fetal development onward and across several brain regions comprising the cortex and subcortex (such as the thalamus, striatum, amygdala, and hippocampus). Expression remained stable in the cortex from early-/mid-fetal stages through young adulthood. However, expression was reduced in the hippocampus, striatum, and thalamus during early adulthood (Fig. 5b and online Supplementary File 3).

### Cell-type-specific expression and cortical layer enrichment analyses

The CSEA highlighted gene set overexpression in three distinct human brain cell types. Specifically, genes related to alterations in cortical thickness depicted significant enrichment in neurons (corrected *p*-value = 6.33 × 10^−7^), oligodendrocytes (corrected *p*-value = 5.21 × 10^−5^), and astrocytes (corrected *p*-value = 0.0012, Fig. 5c). Furthermore, the cortical layer enrichment analysis depicted that these genes were significantly enriched across layers I, II, III, IV, and VI of the cortices, with particularly substantial enrichment within layers II and VI (Fig. 5d).

### PPI analysis and hub genes

**Figure 6.**
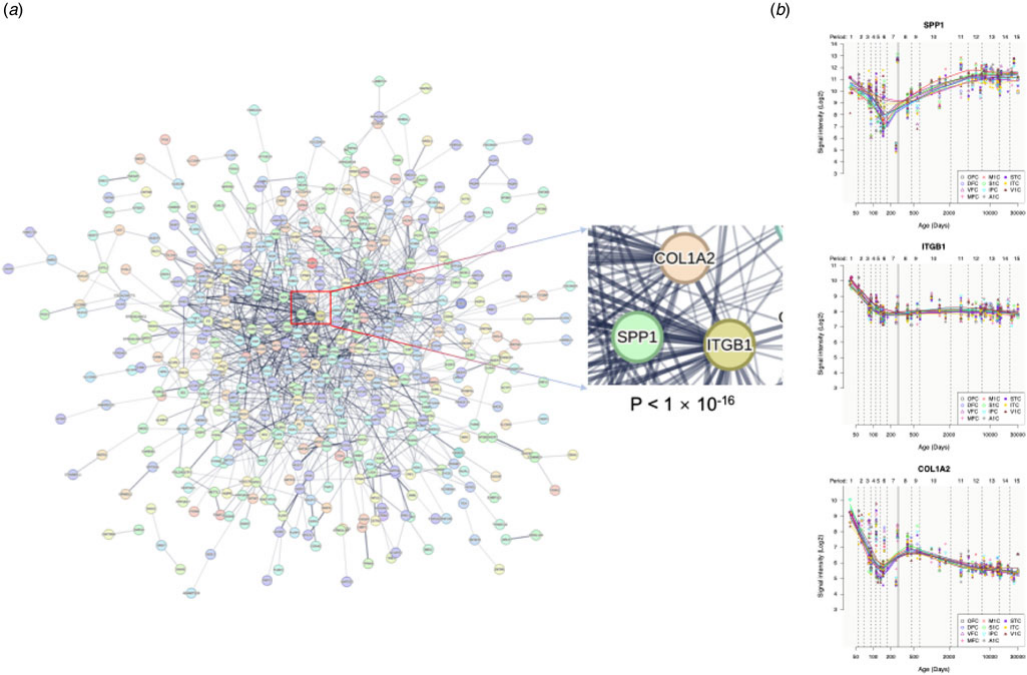
Protein–protein interaction networks and expression patterns of hub genes. (*a*) The whole PPI network with 598 genes and 1137 edges. Each node represents a protein, and each edge represents an interaction between two proteins. The *p* value denotes the statistical significance of how likely the proteins encoded by the input genes are connected to construct a network. (*b*) Spatial–temporal expression curves of three hub genes (i.e. SPP1, ITGB1, and COL1A2). COL1A2, collagen type I alpha 2 chain; PPI, protein–protein interaction; ITGB1, integrin subunit beta 1; SPP1; secreted phosphoprotein 1.

Under an interaction confidence score of 0.4, a network comprising 598 nodes and 1137 edges was constructed. This network contained 809 edges, significantly more than expected by chance (*p* = 1.0 × 10^−16^, Fig. 6a). The top three hub genes in the PPI network were identified with the maximal clique centrality method in CytoHubba (Chin et al., 2014; Shannon et al., 2003): secreted phosphoprotein 1 (*SPP1*), integrin subunit beta 1 (*ITGB1*), and collagen type I alpha 2 chain (*COL1A2*). The spatiotemporal expression trajectories of these three hub genes were delineated, highlighting how their expression patterns change across brain regions and developmental stages (Fig. 6b).

### Genetic overlap with OCD-related genes

Fisher's exact test indicated significant overlap between the 609 cortical thickness-related genes identified in this study and the 173 OCD-associated genes derived from the intersection of 153 genes from the Obsessive–Compulsive Disorder and 64 genes from the MalaCards databases (Privitera et al., 2015; Rappaport et al., 2017). Thirteen genes overlapped between the two gene sets (odds ratio = 3.19, *p* = 4.33 × 10^−4^).

## Discussion

Although OCD is a highly heritable disorder, genome-wide association studies of OCD have identified only one genetic locus reaching genome-wide significance because of limited statistical power to detect genetic variants with subtle effects (Strom et al., 2021). Therefore, the genes implicated in OCD-related brain structure alterations remain obscure. Hence, the present integrative analysis linking cortical thickness changes and transcriptomic data provides novel insights into the genetic and molecular factors of brain abnormalities in OCD. Through spatial correlation analysis, over 1000 differentially expressed genes linked with OCD cortical alterations were identified across two datasets. Further investigation of the 609 overlapping genes highlighted enriched BPs, developmental trajectories, cell-type specificities, and protein interactions contributing to OCD pathogenesis.

The significant overlap of genes between the datasets is a notable discovery, mainly because only a modest spatial correlation of cortical thickness changes was observed between these datasets. The multi-site ENIGMA study is the most extensive investigation of structural morphometry in OCD and provides robust evidence for syndrome-specific cortical thickness abnormalities (Boedhoe et al., 2018). Challenges such as small sample sizes in independent datasets, the confounding effects of medication, and variations in disease severity among participants contribute to this low-spatial correlation. Nonetheless, the consistency of gene association with OCD cortical pathology across different cohorts underscores the robustness of these implicated genes despite the varying patterns of alterations.

It is well-established that genetic factors significantly influence cortical thickness (Jha et al., 2018; Panizzon et al., 2009). These genetic underpinnings intersect with various cellular processes to orchestrate the dynamic development of the cerebral cortex (Grasby et al., 2020; Rakic, 2009). The observed gene overlap indicates that macroscale heterogeneity in OCD neuroimaging findings may converge onto shared molecular pathways. The biological functions and pathways enriched in the gene set provide further insight into the potential mechanisms underlying OCD-related cortical thickness alterations. Notably, the enrichment of genes involved in ion transport regulation, responses to environmental stimuli, and metal ion transport regulation suggests the roles of these processes in OCD pathophysiology. Enrichment in glutamate signaling pathways aligns with evidence that genetic disruptions can lead to circuit-level excitatory/inhibitory imbalance (Ahmari, Risbrough, Geyer, & Simpson, 2012; Hoenig, Hochrein, Quednow, Maier, & Wagner, 2005). Glutamate-mediated excitotoxicity may lead to the structural and functional cortical disturbances observed in OCD (Robbins et al., 2019). Genes involved in synaptic structure and transmission were also overrepresented, consistent with synaptic dysfunction in OCD (Piantadosi et al., 2021; Wang, Kavalali, & Monteggia, 2022). The enriched phospholipase D signaling pathway regulates synaptic function and plasticity (Bruntz, Lindsley, & Brown, 2014). Despite the distinct spatial patterns, this convergence emphasizes the need to connect multi-scale perspectives to elucidate the complex OCD origins.

The tissue-specific and temporal-specific expression analyses of the identified genes highlight roles in brain development and function, supporting the observed transcriptome–neuroimaging relationships. The specific expression of these genes in brain tissue and their expression from early-/mid-fetal development onward underscore their neurodevelopmental process relevance. Stable expression in the cortex during young adulthood but variable expression in other brain regions provides a potential link between the genes and maturation of brain regions implicated in OCD. Enriched expression of cortical thickness-related genes in neurons, oligodendrocytes, and astrocytes connects these specific cell types in OCD pathogenesis (Soto et al., 2023; Writing Committee for the Attention-Deficit/Hyperactivity Disorder et al., 2021). The findings of layer-specific enrichment (particularly layers II and VI of the cortices) suggest a potential role in neural circuitry and connectivity. Deciphering how these genes affect the architecture of cortical layers may provide insights into neural circuits linked with OCD symptomatology (Goodman et al., 2021; Maia, Cooney, & Peterson, 2008).

The PPI network revealed interconnections among the genes. Identifying hub genes, viz., *SPP1*, *ITGB1*, and *COL1A2*, underscores their centrality in the genetic network linked with cortical thickness alterations in OCD. The spatiotemporal expression trajectories of these hub genes provide insights into their dynamic roles across brain regions and developmental stages. Notably, hub gene *SPP1* depicts declining expression from infancy onward, consistent with its hypothesized role in neurodevelopment (Khodosevich & Sellgren, 2023). Type I collagen, produced by genes like *COL1A2*, contributes to the structural integrity of perineuronal net (Fawcett, Oohashi, & Pizzorusso, 2019). These structures are essential for maintaining the physical and biochemical milieu around neurons, offering protection against oxidative stress and regulating synaptic functions (Fawcett et al., 2019). Alterations in COL1A2 expression or activity could potentially contribute to structural or functional changes in the brain relevant to OCD. The significant overlap between the genes related to cortical thickness alterations and the genes associated with OCD (13 overlapping genes) highlights a potential genetic link between the observed structural changes in OCD and the disorder itself. The *SHANK2* gene encodes a protein that regulates synaptic architecture and is linked with neurodevelopmental processes (Lim et al., 1999). Dysfunctional synaptic connectivity and signaling, potentially arising from *SHANK2* variations, could underlie the morphological brain changes observed within OCD patients, particularly in regions linked with OCD symptomatology (Monteiro & Feng, 2017; Radua, van den Heuvel, Surguladze, & Mataix-Cols, 2010). Understanding the shared genetic basis between cortical thickness alterations and OCD could provide more targeted and personalized treatment approaches.

Lastly, some limitations of this study must be noted. First, the gene expression data used in AHBA were obtained from six adult donors and not from psychiatric patients with OCD symptoms. Thus, data from OCD patients must be collected to improve our understanding of psychiatric disorders at the molecular level. Second, most OCD patients in the ENIGMA and independent datasets were chronic cases taking medication, leading to confounding variables related to disease profile and drug use. Future research should include drug-naïve first-episode patients to eliminate these confounding factors and corroborate the initial findings. Moreover, our analysis lacks longitudinal data to determine causality between the implicated genes and cortical changes.

## Conclusion

This study identified over 600 genes related to cortical thickness changes in OCD by integrating neuroimaging and transcriptional data. Subsequent analyses provided insights into the BPs, cell types, and pathways implicated in OCD cortical pathology. These findings bridge the gap between macroscopic modifications in brain structure in OCD and specific microscopic molecular events, thereby providing unique insights into genetic and molecular factors that may contribute to cortical abnormalities in OCD. These findings provide the groundwork for future work to elucidate OCD etiology, develop molecular biomarkers, and identify therapeutic targets.

## Supporting information

Zhang et al. supplementary material 1 Zhang et al. supplementary material

Zhang et al. supplementary material 2 Zhang et al. supplementary material

Zhang et al. supplementary material 3 Zhang et al. supplementary material

Zhang et al. supplementary material 4 Zhang et al. supplementary material

## Data Availability

The findings of this study are supported by a range of valuable resources and tools. Human gene expression data are available from the Allen Brain Atlas (https://human.brain-map.org/static/download) and gene expression analysis was conducted using code available on GitHub (https://github.com/BMHLab/AHBAprocessing). Probe-to-gene annotations were obtained using the Re-annotator toolkit (http://sourceforge.net/projects/reannotator), and preprocessing of neuroimaging data was performed using freely available software (http://rfmri.org/DPABISurf). The code used for PLS analysis is openly available on GitHub (https://github.com/SarahMorgan/Morphometric_Similarity_SZ). Biological function enrichment analysis was performed using the WebGestalt online tool (https://www.webgestalt.org/). Tissue-specific and temporal-specific expression analyses can be conducted using specific websites (http://genetics.wustl.edu/jdlab/tsea/ and http://genetics.wustl.edu/jdlab/csea). Cell-specific gene expression data can be downloaded from the CellMarker database (http://xteam.xbio.top/CellMarker/). Layer markers were obtained from a dataset by He et al. (https://static-content.springer.com/esm/art%3A10.1038%2Fnn.4548/MediaObjects/41593_2017_BFnn4548_MOESM255_ESM.xlsx). Construction of the PPI network utilized the STRING database version 12.0 (https://string-db.org/).

## Supplementary material

The supplementary material for this article can be found at https://doi.org/10.1017/S0033291724001909.
