# Genetic and molecular correlates of cortical thickness alterations in adults with obsessive–compulsive disorder: a transcription–neuroimaging association analysis – CORRIGENDUM

**DOI:** 10.1017/S003329172400271X

**Published:** 2024-10

**Authors:** Da Zhang, Changjun Teng, Yinhao Xu, Lei Tian, Ping Cao, Xiao Wang, Zonghong Li, Chengbin Guan, Xiao Hu

The original version of this article was published with incorrect corresponding author information.

This has now been amended.

## References

[ref1] Zhang, D., Teng, C., Xu, Y, et al. Genetic and molecular correlates of cortical thickness alterations in adults with obsessive–compulsive disorder: a transcription–neuroimaging association analysis. Psychological Medicine. Published online 2024:1–10. doi:10.1017/S0033291724001909 PMC11496223 39363543

