# Supplementary material for: Genetic and molecular correlates of cortical thickness alterations in adults with obsessive–compulsive disorder: a transcription–neuroimaging association analysis
Source: Psychol Med. 2024 Oct 4;54(12):3469–78. doi: 10.1017/S0033291724001909 (PMC11496223; doi:10.1017/S0033291724001909)
Supplement: Zhang et al. supplementary material 4 — Zhang et al. supplementary material [file S0033291724001909sup004.docx]

**Supplementary materials**

# Materials and methods

**1.1 Participants in the independent dataset**

Following the appropriate ethics approval, this study was conducted at the Affiliated Brain Hospital of Nanjing Medical University. A total of 76 OCD patients and 75 healthy controls were recruited, all of whom provided written informed consent. The inclusion criteria for the case group were as follows: OCD diagnosis by a professional psychiatrist based on the Chinese Classification of Mental Disorders (third edition), a total score on the Yale Brown Obsessive Compulsive Scale (Y-BOCS) ≥ 16, no history of other psychiatric disorder, no history of organic brain disease, no history of electroconvulsive and severe somatic disease, right-handedness, and normal vision or corrected vision. Healthy controls were college or high school students who volunteered to participate and did not exhibit any history of serious medical or neuropsychiatric illness; furthermore, HCs had no known history of psychiatric illness among their first-degree relatives. Exclusion criteria for all participants included: (a) age younger than 18 years or older than 60 years; (b) any history of OCD, neurodevelopmental, affective or psychotic illness; (c) significant systemic or neurologic illness or neurosurgery; (d) history of substance abuse or dependence disorders; and (e) pregnancy and other MR contraindications. The severity of subjects’ obsessive-compulsive symptomatology was measured using the 10-item clinician-rated Yale- Brown Obsessive Compulsive Scale (Y-BOCS)(Goodman et al., 1989).

**1.2 MRI data acquisition in the independent dataset**

Multi-contrast MRI data were acquired at Nanjing Brain Hospital, China, using a Siemens 3.0T Discovery MR750 scanner (GE Healthcare). Foam padding was utilized to mitigate head motion, and participants were instructed to keep their eyes closed and head stationary throughout the scanning session.

Structural MRI data were acquired using a magnetization-prepared rapid gradient-echo sequence with the following parameters: TR (Repetition Time): 8.2 ms, TE (Echo Time): 3.2 ms, flip angle: 12°, FOV (Field of View) frequency=256 mm, FOV phase=10 mm, matrix size: 256×256, slice thickness: 1 mm, interslice gap: 1 mm, and a total of 172 slices.

Resting-state functional MRI data were acquired using an echo-planar imaging sequence with the following parameters: TR/TE: 2000/30 ms, flip angle: 90°, 40 transverse slices, FOV frequency=100 mm, FOV phase=10 mm, matrix size: 64×64, slice thickness: 4 mm, interslice gap: 4 mm, aligned along the anterior commissure-posterior commissure line, resulting in a total of 9600 volumes.

**1.3 Image processing in the independent dataset**

The structural images underwent preprocessing using a surf-based resting-state fMRI data analysis toolbox (DPABISurf 1.8)(Yan, Wang, & Lu, 2021) in MATLAB R2019a. In brief, the processing of the structural image encompassed skull stripping, cortical extraction, segmentation of cortical white and gray matter, separation of the hemisphere and subcortical structures, and surface reconstruction of the gray/white interface and the pial surface(Dale, Fischl, & Sereno, 1999)(Y. Zhang, Brady, & Smith, 2001)(Avants, Epstein, Grossman, & Gee, 2008)(Klein et al., 2017). The T1-weighted image was subjected to correction for intensity non-uniformity (INU) utilizing the N4BiasFieldCorrection algorithm and served as the T1w reference throughout the workflow(Tustison et al., 2010). Images with significant organic abnormalities were excluded. The cortical thickness was calculated for 360 regions of the Human Connectome Project (HCP) multi-modal parcellation 1.0 atlas (180 in each hemisphere)(Glasser et al., 2016). Cortical thickness were corrected for age and sex, the values for maps are z-scored effect sizes (Cohen’s d) of cortical thickness in patient populations versus healthy controls.

1.4 Details of enrichment analyses

Genetic Overlaps with OCD-Related Genes

To explore the overlap with established OCD genetics, we conducted a comparative analysis of genes associated with changes in cortical thickness in OCD patients (identified in this study) with OCD-related genes compiled in the MalaCards database (https://www.malacards.org/) and the Obsessive-Compulsive Disorder Database (http://alpha.dmi.unict.it/ocdb/)(Rappaport et al., 2017)(Privitera et al., 2015). Significance was assessed using Fisher's exact test with the reference gene set consisting of the re-annotated 20,737 genes expressed in the brain across the entire genome (utilized as the background gene set for all enrichment analyses)(Fisher, 1922)(Arloth, Bader, Röh, & Altmann, 2015).

Enrichment for biological functions

WebGestalt (WEB-based Gene SeT AnaLysis Toolkit) is a functional enrichment analysis web tool that garners an average of 26,000 unique users from 144 countries and territories annually, as per Google Analytics(Liao, Wang, Jaehnig, Shi, & Zhang, 2019). Gene Ontology (GO) was employed to ascertain the biological functions, encompassing Molecular Functions (MFs), Biological Processes (BPs), and Cellular Components (CCs)(Thomas et al., 2022). The Kyoto Encyclopedia of Genes and Genomes (KEGG) was utilized to identify related biological pathways(Kanehisa, Furumichi, Tanabe, Sato, & Morishima, 2017). The enrichment results obtained were thresholded for significance at 5%, corrected by the Benjamini and Hochberg method for False Discovery Rate (BH-FDR)(“Controlling the False Discovery Rate: A Practical and Powerful Approach to Multiple Testing - Benjamini - 1995 - Journal of the Royal Statistical Society: Series B (Methodological) - Wiley Online Library,” n.d.).

Tissue-Specific Expression Analysis and Temporal-specific expression analysis

The online tools for Tissue-Specific Expression Analysis (TSEA) and Cell-Specific Expression Analysis (CSEA) (http://genetics.wustl.edu/jdlab/tsea/ and http://genetics.wustl.edu/jdlab/csea/) were employed to perform specialized analyses regarding tissue and temporal expressions(Dougherty, Schmidt, Nakajima, & Heintz, 2010)(Xu, Wells, O’Brien, Nehorai, & Dougherty, 2014). The Specificity Index Threshold (pSI) package(Xu et al., 2014) was employed to elucidate the distinct expression patterns of two gene sets across the lifespan of an organism, encompassing six major brain regions (i.e., cortex, thalamus, striatum, cerebellum, hippocampus, amygdala) throughout 10 developmental periods (from early fetal to young adulthood). Significant intervals were discerned using Fisher's exact test at a pSI threshold of 0.05, followed by the application of the Bonferroni correction to adjust for the number of gene sets and stages (p < 0.05). Furthermore, this package facilitated the evaluation of gene enrichment in specific tissues in comparison to others, employing a pSI threshold of 0.05. This approach provides a comprehensive understanding of gene enrichment and expression patterns at different stages of an organism’s life.

Cell type-specific expression and cortical layer enrichment analysis

Since the brain consists of various types of cells with specific gene expression, cell type-specific analysis was performed to explore the specific cell types associated with the significant PLS1+/- gene sets(Dougherty et al., 2010). Cell type-specific gene expression data of the human brain neurons, astrocytes, oligodendrocytes, microglia, and macrophage were downloaded from the CellMarker database(X. Zhang et al., 2019). We conducted a cortical layer enrichment analysis utilizing layer-specific marker genes that were identified from prior single-cell transcriptomic profiling(He et al., 2017). The marker gene sets contained 772 genes for layer I, 483 for layer II, 294 for layer III, 308 for layer IV, 115 for layer V, and 2,159 for layer VI. Fisher's exact test was used to identify in which cell type or cortical layer each gene set was overexpressed, followed by Bonferroni correction for the numbers of gene sets and cell types (*p* < 0.05)(Curtin & Schulz, 1998).

Protein-protein interaction analysis

Protein-protein interaction (PPI) analysis was performed with STRING v12.0 (https://string-db.org/) to determine whether genes linked to cortical thickness changes in OCD could assemble into a biologically meaningful PPI network using a medium confidence value of 0.4(Szklarczyk et al., 2023). Key nodes within the Protein-Protein Interaction (PPI) network were examined using the cytoHubba plugin in Cytoscape software (version 3.10.1)(Chin et al., 2014)(Shannon et al., 2003). CytoHubba offers various topological algorithms to identify important nodes within a network. Among these algorithms, Maximal Clique Centrality (MCC) is often considered the most effective(Chin et al., 2014). Thus, the MCC method was employed to pinpoint the top three essential genes in the PPI network, which are regarded as hub genes with significant biological functions. The Human Brain Transcriptome Atlas (http://hbatlas.org/) was leveraged to map the spatial and temporal expression trajectories of the hub genes exhibiting maximal connectivity(Pletikos et al., 2014).

# Tables and Figures

**Table.S1** Demographic and clinical characteristics of patients with Obsessive–compulsive disorder (OCD) and healthy controls (HCs).

| Characteristic | | | |  | OCD^a^ (n=57) | | | | | | HC (n=57) | | | | | | P-value | | | |  |
| --- | --- | --- | --- | --- | --- | --- | --- | --- | --- | --- | --- | --- | --- | --- | --- | --- | --- | --- | --- | --- | --- |
|  |  |  |  |  | Mean | | | SD | | | Mean | | | | SD | |  |  |  |  |  |
|  | | Ages,years | |  | 28.28 | | | 9.04 | | | 26.11 | | | | 5.48 | | 0.124^c^ | | | |  |
|  | | Sex(male/female) | | | 29/28 | | | NA | | | 37/20 | | | | NA | | 0.129^b^ | | | |  |
|  | | Education | |  | 15.07 | | | 1.45 | | | 16.79 | | | | 2.61 | | <0.001^c^ | | | |  |
|  | | Y-BOCS | |  |  |  | | |  |  | | |  | | |  | | |  |  | |
|  | | Total | | 26.21 | | 5.71 | | | | | NA | | NA | | | | NA | | | | |
|  | | Obessive subscale score | | 13.98 | | 4.15 | | | | | NA | | NA | | | | NA | | | | |
|  | | Compulsive subscale score | | 12 | | 2.99 | | | | | NA | | NA | | | | NA | | | | |

^a^Thirty-four patients were drug-naïve, and twenty-three patients were medicated with SSRI for more than 12 weeks.

^b^Two-tailed χ 2-tests.

^c^Two-tailed t-tests.

Abbreviations: NA, not available/not applicable; SD, standard deviation; SSRI, selective serotonin reuptake inhibitor Y-BOCS, Yale-Brown Obsessive Compulsive Scale.

**Table.S2** Demographic information of the six adult donors in the AHBA.

| Donor | Age (years) | Gender | Ethnicity | Hemisphere | Post-mortem interval (h) |
| --- | --- | --- | --- | --- | --- |
| H0351.2001 | 24 | Male | African American | Both | 23 |
| H0351.2002 | 39 | Male | African American | Both | 10 |
| H0351.1009 | 57 | Male | Caucasian | Left | 25.5 |
| H0351.1012 | 31 | Male | Caucasian | Left | 17.5 |
| H0351.1015 | 49 | Female | Hispanic | Left | 30 |
| H0351.1016 | 55 | Male | Caucasian | Left | 18 |

Abbreviations: AHBA, Allen Human Brain Atlas.

**Figure. S1** The remaining probes and genes at each processing step.

# References

Arloth, J., Bader, D. M., Röh, S., & Altmann, A. (2015). Re-Annotator: Annotation Pipeline for Microarray Probe Sequences. *PloS One*, *10*(10), e0139516. https://doi.org/10.1371/journal.pone.0139516

Avants, B. B., Epstein, C. L., Grossman, M., & Gee, J. C. (2008). Symmetric diffeomorphic image registration with cross-correlation: Evaluating automated labeling of elderly and neurodegenerative brain. *Medical Image Analysis*, *12*(1), 26–41. https://doi.org/10.1016/j.media.2007.06.004

Chin, C.-H., Chen, S.-H., Wu, H.-H., Ho, C.-W., Ko, M.-T., & Lin, C.-Y. (2014). cytoHubba: Identifying hub objects and sub-networks from complex interactome. *BMC Systems Biology*, *8 Suppl 4*(Suppl 4), S11. https://doi.org/10.1186/1752-0509-8-S4-S11

Controlling the False Discovery Rate: A Practical and Powerful Approach to Multiple Testing—Benjamini—1995—Journal of the Royal Statistical Society: Series B (Methodological)—Wiley Online Library. (n.d.). Retrieved October 19, 2023, from https://rss.onlinelibrary.wiley.com/doi/abs/10.1111/j.2517-6161.1995.tb02031.x

Curtin, F., & Schulz, P. (1998). Multiple correlations and Bonferroni’s correction. *Biological Psychiatry*, *44*(8), 775–777. https://doi.org/10.1016/s0006-3223(98)00043-2

Dale, A. M., Fischl, B., & Sereno, M. I. (1999). Cortical surface-based analysis. I. Segmentation and surface reconstruction. *NeuroImage*, *9*(2), 179–194. https://doi.org/10.1006/nimg.1998.0395

Dougherty, J. D., Schmidt, E. F., Nakajima, M., & Heintz, N. (2010). Analytical approaches to RNA profiling data for the identification of genes enriched in specific cells. *Nucleic Acids Research*, *38*(13), 4218–4230. https://doi.org/10.1093/nar/gkq130

Fisher, R. A. (1922). On the Interpretation of χ2 from Contingency Tables, and the Calculation of P. *Journal of the Royal Statistical Society*, *85*(1), 87–94. https://doi.org/10.2307/2340521

Glasser, M. F., Coalson, T. S., Robinson, E. C., Hacker, C. D., Harwell, J., Yacoub, E., … Van Essen, D. C. (2016). A multi-modal parcellation of human cerebral cortex. *Nature*, *536*(7615), 171–178. https://doi.org/10.1038/nature18933

Goodman, W. K., Price, L. H., Rasmussen, S. A., Mazure, C., Fleischmann, R. L., Hill, C. L., … Charney, D. S. (1989). The Yale-Brown Obsessive Compulsive Scale: I. Development, Use, and Reliability. *Archives of General Psychiatry*, *46*(11), 1006–1011. https://doi.org/10.1001/archpsyc.1989.01810110048007

He, Z., Han, D., Efimova, O., Guijarro, P., Yu, Q., Oleksiak, A., … Khaitovich, P. (2017). Comprehensive transcriptome analysis of neocortical layers in humans, chimpanzees and macaques. *Nature Neuroscience*, *20*(6), 886–895. https://doi.org/10.1038/nn.4548

Kanehisa, M., Furumichi, M., Tanabe, M., Sato, Y., & Morishima, K. (2017). KEGG: New perspectives on genomes, pathways, diseases and drugs. *Nucleic Acids Research*, *45*(D1), D353–D361. https://doi.org/10.1093/nar/gkw1092

Klein, A., Ghosh, S. S., Bao, F. S., Giard, J., Häme, Y., Stavsky, E., … Keshavan, A. (2017). Mindboggling morphometry of human brains. *PLoS Computational Biology*, *13*(2), e1005350. https://doi.org/10.1371/journal.pcbi.1005350

Liao, Y., Wang, J., Jaehnig, E. J., Shi, Z., & Zhang, B. (2019). WebGestalt 2019: Gene set analysis toolkit with revamped UIs and APIs. *Nucleic Acids Research*, *47*(W1), W199–W205. https://doi.org/10.1093/nar/gkz401

Pletikos, M., Sousa, A. M. M., Sedmak, G., Meyer, K. A., Zhu, Y., Cheng, F., … Sestan, N. (2014). Temporal specification and bilaterality of human neocortical topographic gene expression. *Neuron*, *81*(2), 321–332. https://doi.org/10.1016/j.neuron.2013.11.018

Privitera, A. P., Distefano, R., Wefer, H. A., Ferro, A., Pulvirenti, A., & Giugno, R. (2015). OCDB: A database collecting genes, miRNAs and drugs for obsessive-compulsive disorder. *Database: The Journal of Biological Databases and Curation*, *2015*, bav069. https://doi.org/10.1093/database/bav069

Rappaport, N., Twik, M., Plaschkes, I., Nudel, R., Iny Stein, T., Levitt, J., … Lancet, D. (2017). MalaCards: An amalgamated human disease compendium with diverse clinical and genetic annotation and structured search. *Nucleic Acids Research*, *45*(D1), D877–D887. https://doi.org/10.1093/nar/gkw1012

Shannon, P., Markiel, A., Ozier, O., Baliga, N. S., Wang, J. T., Ramage, D., … Ideker, T. (2003). Cytoscape: A software environment for integrated models of biomolecular interaction networks. *Genome Research*, *13*(11), 2498–2504. https://doi.org/10.1101/gr.1239303

Szklarczyk, D., Kirsch, R., Koutrouli, M., Nastou, K., Mehryary, F., Hachilif, R., … von Mering, C. (2023). The STRING database in 2023: Protein-protein association networks and functional enrichment analyses for any sequenced genome of interest. *Nucleic Acids Research*, *51*(D1), D638–D646. https://doi.org/10.1093/nar/gkac1000

Thomas, P. D., Ebert, D., Muruganujan, A., Mushayahama, T., Albou, L., & Mi, H. (2022). PANTHER: Making genome‐scale phylogenetics accessible to all. *Protein Science : A Publication of the Protein Society*, *31*(1), 8–22. https://doi.org/10.1002/pro.4218

Tustison, N. J., Avants, B. B., Cook, P. A., Zheng, Y., Egan, A., Yushkevich, P. A., & Gee, J. C. (2010). N4ITK: Improved N3 bias correction. *IEEE Transactions on Medical Imaging*, *29*(6), 1310–1320. https://doi.org/10.1109/TMI.2010.2046908

Xu, X., Wells, A. B., O’Brien, D. R., Nehorai, A., & Dougherty, J. D. (2014). Cell type-specific expression analysis to identify putative cellular mechanisms for neurogenetic disorders. *The Journal of Neuroscience: The Official Journal of the Society for Neuroscience*, *34*(4), 1420–1431. https://doi.org/10.1523/JNEUROSCI.4488-13.2014

Yan, C.-G., Wang, X.-D., & Lu, B. (2021). DPABISurf: Data processing & analysis for brain imaging on surface. *Science Bulletin*, *66*(24), 2453–2455. https://doi.org/10.1016/j.scib.2021.09.016

Zhang, X., Lan, Y., Xu, J., Quan, F., Zhao, E., Deng, C., … Xiao, Y. (2019). CellMarker: A manually curated resource of cell markers in human and mouse. *Nucleic Acids Research*, *47*(D1), D721–D728. https://doi.org/10.1093/nar/gky900

Zhang, Y., Brady, M., & Smith, S. (2001). Segmentation of brain MR images through a hidden Markov random field model and the expectation-maximization algorithm. *IEEE Transactions on Medical Imaging*, *20*(1), 45–57. https://doi.org/10.1109/42.906424
